# Combination therapy of PKCζ and COX-2 inhibitors synergistically suppress melanoma metastasis

**DOI:** 10.1186/s13046-017-0585-2

**Published:** 2017-09-02

**Authors:** Ping Zhou, Jiaqi Qin, Yuan Li, Guoxia Li, Yinsong Wang, Ning Zhang, Peng Chen, Chunyu Li

**Affiliations:** Department of Thoracic Medical Oncology, Tianjin Medical University Cancer Institute and Hospital, School of Basic Medical Sciences, International Medical School, School of Pharmacy, Tianjin Medical University, No. 22 Qixiangtai Road, Heping District, Tianjin, 300070 People’s Republic of China

**Keywords:** PKCζ inhibitor, Cox-2, Celecoxib, Melanoma metastasis, Combination therapy

## Abstract

**Background:**

Metastatic malignant melanoma is one of the most aggressive malignancies and its treatment remains challenging. Recent studies demonstrate that the melanoma metastasis has correlations with the heightened activations of protein kinase C ζ (PKCζ) and cyclooxygenase-2 (COX-2) signaling pathways. Targeted inhibitions for PKCζ and COX-2 have been considered as the promising strategies for the treatment of melanoma metastasis. Thus, the PKCζ inhibitor J-4 and COX-2 inhibitor Celecoxib were combined to treat melanoma metastasis in this study.

**Methods:**

The Transwell assay, Wound-healing assay and Adhesion assay were used to evaluate the inhibition of combined therapy of J-4 and Celecoxib on melanoma cells invasion, migration and adhesion in vitro, respectively. The impaired actin polymerization was observed by confocal microscope and inactivated signal pathways about PKCζ and COX-2 were confirmed by the Western blotting assay. The B16-F10/C57BL mouse melanoma model was used to test the inhibition of combined therapy of J-4 and Celecoxib on melanoma metastasis in vivo.

**Results:**

The in vitro results showed that the combination of J-4 and Celecoxib exerted synergistic inhibitory effects on the migration, invasion and adhesion of melanoma B16-F10 and A375 cells with combination index less than 1. The actin polymerization and phosphorylation of Cofilin required in cell migration were severely impaired, which is due to the inactivation of PKCζ related signal pathways and the decrease of COX-2. The combined inhibition of PKCζ and COX-2 induced Mesenchymal-Epithelial Transition (MET) in melanoma cells with the expression of E-Cadherin increasing and Vimentin decreasing. The secretion of MMP-2/MMP-9 also significantly decreased after the combination treatment. In C57BL/6 mice intravenously injected with B16-F10 cells (5 × 10^4^ cells/mouse), co-treatment of J-4 and Celecoxib also severely suppressed melanoma lung metastasis. The body weight monitoring and HE staining results indicated the low toxicity of the combination therapy.

**Conclusions:**

This study demonstrates that the combination therapy of PKCζ and COX-2 inhibitors can significantly inhibit melanoma metastasis in vitro and in vivo, which will be an efficient strategy for treatment of melanoma metastasis in clinics.

## Background

Melanoma is a most aggressive and lethal form of skin cancer. The incidence of melanoma continues to increase and is always accompanied with poor survival worldwide [1, 2]. The treatment of malignant melanoma, especially advanced and metastasized melanoma, remains high challenging due to its extensive metastasis, fast progression and limited effective drugs [3]. There has been no indicated treatment to affect the disease’s outcome until now. Although adoptive cancer immunotherapy with transgenic T cell receptor engineered anti-tumor T cells has produced encouraging results, the efficacy of these approaches has to be improved [4]. Thus, it is very urgent to develop an effective treatment for inhibiting melanoma metastasis. Recent studies have evidenced the reasonability of drug combinations as a promising strategy for melanoma treatment in preliminary [5, 6].

Elevated expression of COX-2 is a common characteristic of many human carcinomas. COX-2 plays an important role in tumorigenesis as mediating the progression and metastasis of tumors, such as nasopharyngeal carcinoma [7], hepatocellular carcinoma [8], lung cancer [9] and melanoma [10]. The differential expression of COX-2 highly correlates to the progression of malignant melanoma [11] and severely impairs the survival of patients [12]. As previous reports, COX-2 regulates membrane permeability of B16-F10 cells via cPLA2 [13] and COX-2 related signaling pathways have been confirmed involved in melanoma metastasis [10, 14]. Celecoxib, as a highly selective COX-2 inhibitor, has been tested in many clinical trials, including pancreatic cancer [15], nonmelanoma skin cancer [16] and colorectal cancer [17]. Celecoxib exhibits significant antitumor effects in COX-2 expressing and non-expressing melanoma cell lines through inducing apoptosis or inhibiting migration [18]. The combination of Celecoxib with other drugs represents a new standard for melanoma treatment [5]. For example, Celecoxib could enhance the inhibition of melanoma growth and metastasis by dacarbazine [19]; Celecoxib and plumbagin shows synergistic inhibitory effects on melanoma tumor growth [20]. In recent studies, targeted therapy with BRAF inhibitors displayed modest antitumor activity and amplified the pro-apoptotic activity of MEK inhibitors by inducing ER stress in NRAS-mutant melanoma [21]. However, BRAF inhibitors were reperted to accelerated skin tumors and soft agar colonies in DMBA/TPA tumor induction. Celecoxib significantly delayed tumor acceleration by the BRAF inhibitor PLX7420 or vemurafenib. MEK inhibitor, trametinib, also reduced vemurafenib-induced PDV soft agar colonies, but less efficiently than celecoxib [22].

In our previous studies, PKCζ, an atypical protein kinase C, functioned as a crucial mediator in chemotaxis of macrophages [23] and various cancer cells, such as human breast cancer cells [24, 25], glioblastoma cells [26] and lung cancer cells [27]. Briefly, PKCζ is required for EGF-induced chemotaxis and regulates actin polymerization and cell adhesion via involved in PI3K/Akt pathway and affecting phosphorylation of LIMK and Cofilin. The expression of PKCζ is commonly elevated in human and murine melanoma cells than melanocytes [28], especially in interferon-resistant cells [29]. The elevated activated PKCζ is mainly involved in metastasis associated signaling pathways in melanoma cells [30]. Besides regulating actin polymerization and cell adhesion as in other carcinoma cells, PKCζ could also regulate melanoma cells invasion via affecting the expression and activities of matrix metallprotease-1, −2, −9 and MT1-MMP [31]. In addition, Collagen induced nuclear translocation of NF-κB is dependent on PKCζ pathway, which is essential for migration [32]. Some small inhibitors specific for PKCζ screened by our group have exhibited great capability in inhibiting breast cancer metastasis [33, 34], among which J-4 is a highly selective inhibitor of PKCζ with inhibitory IC_50_ at approximately 10 μM [35]. J-4 severely impairs cell migration without affecting proliferation, probably because of PKCζ not involved in cell survival dependent signal pathways. Due to its prominent inhibition on metastasis and low toxicity, J-4 has been tested in preclinical studies by the Pharmaceutical Research Center of Tianjin Cancer Institute and Hospital. Therefore, we hypothesized that combined inhibition of PKCζ and COX-2 by J-4 and Celecoxib would synergistically block melanoma metastasis both in vitro and in vivo.

## Methods

### Reagents and antibodies

J-4 was acquired from Maybridge Chemical (Cambrige, CBS, UK). Celecoxib was purchased from Meilun Biological Technology (Dalian, China). Antibodies against Vimentin (AF7013), COX-2 (AF7003) and β-actin (T0022) were purchased from Affinity Biosciences (Shanghai, China). Antibodies against E-Cadherin (#14472), phospho-Cofilin (#3311), Cofilin (#3312), phospho-PKCζ (#9378) and PKCζ (#9372) were obtained from Cell Signaling Technology (Cambridge, MA, USA). Antibodies against MMP-2 (sc-53,630) and MMP-9 (sc-21,733) were purchased from Santa Cruz Biotechnology (Dallas, TX, USA.). Phosphatase inhibitor Cocktail tablets were purchased from Roche Molecular Biochemicals (Indianapolis, IN, USA). Z’-LYTE™ KINASE ASSAY KIT-SER/THR 7 PEPTIDE kit (Cat. No. PV3180) and PKCζ (Cat. No.2273) were purchased from Invitrogen (Carlsbad, CA, USA). Methylthiazolyldiphenyl-tetrazolium bromide (MTT) was purchased from Sigma-Aldrich (USA). DMEM medium, fetal bovine serum (FBS) and penicillin/streptomycin were all obtained from Gibco (Thermo Fisher Scientific Inc., USA).

### Cell lines and cell culture

Mouse melanoma cell line B16-F10 was purchased from the Cell Culture Center of Chinese Academy of Medical Sciences (Beijing, China) and cultured according to the instructions. Human melanoma cell line A375 was characterized by Genetic Testing Biotechnology Corporation (Suzhou, China) using short tandem repeat (STR) markers. The cells were cultured in DMEM medium containing 10% FBS and penicillin/streptomycin at 37 °C in an atmosphere containing 5% CO_2_.

### Z’-LYTE™ assay

The Z’-LYTE™ assay was carried out according to the manufacturer’s instruction. Briefly, 20 μL/well reactions were set up in 384-well plates containing kinase buffer, 5 μM ATP, 4 μM ZMTP, 4 Ser/Thr peptide substrate, 50 ng/μL PKCζ and J4 with different concentrations (0, 5, 10, 25, 50, 100 μM). After 1-h incubation, the development buffer was added to each well and further reacted for 1 h, and followed by reaction stopping. The fluorescence signal ratio of coumarin at 445 nm and fluorescin at 520 nm was then calculated to evaluate the kinase inhibitory activity of J4 in the reaction.

### MTT assay

MTT assay was used to evaluate the effect of J-4 and Celecoxib on cell proliferation. B16-F10 or A375cells were seeded into 96-well plates at 4000/well, incubated at 37 °C in 5% CO_2_. Then cells were treated with J-4 at various doses, Celecoxib (25 μM) or their combination, respectively, for 24 h. MTT reagent was added to each well for further 4 h incubation. The medium was then discarded, and 150 μl of DMSO was added to each well. Subsequently, the plates were shaken for 30 s and the absorbance of each well was measured at 490 nm using a microplate reader (BioTek Epoch, Winooski, VT, USA).

### Wound-healing assay

Cell motility was measured using the Wound-healing assay according to protocol described previously [35]. Typically, B16-F10 or A375 cells were seeded into 60 mm dishes at a density of 8 × 10^5^cells/well and incubated for 12 h to grow a monolayer. After that, the culture media were replaced with the fresh culture media containing J4 (25 μM) and/or Celecoxib (25 μM), and the cells were further incubated for 24 h. Next, a linear scratch wound was created across the middle of the well surface using a pipette tip. The cells were then incubated in serum-free medium at 37 °C in 5% CO_2_. At predetermined time points (0, 3, 6, 9, 12 and 24 h), the wound widths were quantified and photomicrographs were taken with an IX50 inverted microscope (Olympus, Tokyo, Japan). The experiment was carried out in double blind to eliminate the deviation induced by subjective factors.

### Cell invasion assay

Cell invasion in vitro were evaluated by Transwell assays. B16-F10 or A375 cells were pretreated with J-4 and/or Celecoxib at various doses and then seeded into the upper chamber coated with Matrigel matrix (BD Biosciences, MA, USA) at a density of 3.5 × 10^4^ cells/well in serum-free media containing or not containing various doses of J-4 and/or Celecoxib. The lower chambers were filled with media containing 10% FBS. The cells were allowed to migrate for 24 h incubated at 37 °C in 5% CO_2_. The cells that migrated through the polycarbonate membrane were stained and counted visually in 5 random fields using the computer-based microcopy imaging system. The dose-effect curve and combination index (CI) was calculated by the *CalcuSyn software 2.1*. The experiment was carried out in double blind to eliminate the deviation induced by subjective factors.

### Adhesion assay

Adhesion assay was performed as described previously [34]. Briefly, B16-F10 or A375 cells were treated with J4 (25 μM) and/or Celecoxib (25 μM) for 6 h, trypsinized, and re-suspended in serum-free media at a density of 3 × 10^5^ cells/mL. After incubation for additional 30 min, 1.5 mL of cell suspension was placed in 35 mm dishes containing glass cover slips that were coated with 10 ng/mL fibronectin. After further incubations for 5, 15 and 30 min, the cells were washed, fixed and counted in five separate fields under a light microscope. The experiment was carried out in double blind to eliminate the deviation induced by subjective factors.

### Western blotting assay

Western blotting assay was used for assessment of expressions of COX-2, p-PKCζ and p-Cofilin in B16-F10 and A375 cells. The cells were treated with J4 (25 μM) and/or Celecoxib (25 μM) in serum-containing and serum-free media separately for 12 h, and then stimulated by 20 ng/mL EGF for 10 min before lysed on ice for 30 min. Subsequently, 15 μg of protein per sample were separated by 10% SDS-PAGE systems and transferred onto PVDF membranes. After blocking in 5% fat-free milk for 1 h, the membranes were probed with diluted primary antibodies overnight at 4 °C. The antibodies and dilution factors were as follows: COX-2 (1:500), β-actin (1:3000), p-PKCζ (1:1000), PKCζ (1:3000), p-Cofilin (1:500), Cofilin (1:1000), E-Cadherin (1:1000), Vimentin (1:1000), MMP-2 (1:800) and MMP-9 (1:800). Secondary antibodies conjugated with HRP were incubated for further 1 h at room temperature. A G-BOX (Gene Company Ltd., Beijing, China) was used to photograph and analyze bands using *ImageJ* software.

### F-actin content assay

F-actin was quantified by methanol extraction of Oregon Green 568/phalloidin–stained cells as described previously [24]. Briefly, B16-F10 or A375 cells were plated and cultured for 18 h in complete medium followed by further culturing in serum free medium for 3 h. Cells were then treated with the indicated inhibitors or DMSO for 2 h and stimulated by 50 ng/mL EGF at 37 °C. Cells were fixed, permeabilized, and stained in the dark with Oregon Green 568 phalloidin diluted in F-buffer (10 mM HEPES, 20 mM KH_2_PO_4_, 5 mM EGTA, 2 mM MgCl_2_, PBS, pH 6.8) at room temperature for 60 min. After five washes, bound phalloidin was extracted with methanol at 4 °C and subjected to fluorescence analysis at 578 nm excitation and 600 nm emission. At the same time, an aliquot of cells were analyzed by a bicinchoninic acid assay (Pierce, Thermo Fisher Scientific Inc., USA) to determine total protein in the sample. Fluorescence signals were normalized against total protein. Results were expressed as relative F-actin content, where.

*F-actin Δt / F-actin 0 = (fluorescence Δt / mg/mL) / (fluorescence 0 / mg/mL).*

For observation of F-actin filaments, the cells were fixed and stained with rhodamine phalloidin (14 μM; Cytoskeleton, Denver, USA) in the dark for 30 min and finally imaged using a laser scanning confocal microscope (LSCM) (FV1000; Olympus, Tokyo, Japan).

### Real time PCR (RT-PCR)

Total RNA from cells pretreated with J-4 and/or Celecoxib was extracted by using Trizol. Then, RNA was transcribed by using a FastQuant RT kit (TIANGEN, China). The amplification reaction was carried out for 35 cycles. Each cycle consisted of denaturation for 1 min at 95 °C, annealing for 45 s and an extension for 1 min at 72 °C. A final extension step at 72 °C for 5 min terminated the amplification. The primer sequences as previous reports [23, 36, 37], the predicted amplicon sizes, and the annealing temperatures are depicted in Table 1.

**Table 1:** Primer Sequences and Reaction Properties

Target		Sequence	Product Size (bp)	Annealing temp. (°C)
Human				
PKCζ	forward	CTGAGGAGCACGCCAGGTT	625	58.1
	reverse	ACGGGCTCGCTGGTGAACT		
COX-2	forward	TCTGCAGAGTTGGAAGCA-CTCTA	216	58.4
	reverse	GCCGAGGCTTTTCTACCAGAA		
β-actin	forward	CTGGCACCCAGCACAATG	458	54.1
	reverse	GCCGATCCACACGGAGTACT		
Mouse				
PKCζ	forward	ACGGACAACCCTGACATGAAC	361	57.1
	reverse	ATTCGGACTGGTCGATCCTCT		
COX-2	forward	TCAGGTCATTGGTGGAGAGG	96	54
	reverse	GCAAACTGCAGGTTCTCAGG		
β-actin	forward	ATGGAGCCACCGATCCACA	426	56.2
	reverse	CATCCGTAAAGACCTCTATGCCAAC		

### In vivo study in B16-F10/C57BL mouse melanoma lung metastasis model

C57BL/6 mice, 5–6 weeks old, were purchased from the Food and Drug Verification Institute (Beijing, China). All animal experiments were approved by the Animal Ethics Committee of Tianjin Medical University and complied with its regulations. A mouse model of melanoma lung metastasis was constructed by injection of B16-F10 cells into mice (5 × 10^4^cells/mouse) via tail vein. Compound treatment started the next day after melanoma cells injection. The mice were intravenously injected with normal saline, J-4 (20 mg/kg), Celecoxib (20 mg/kg), or their combination every three days, respectively. Mice were sacrificed after 3-week treatment, and the lungs were separated to examine the number of lung metastasis nodules. Then the lungs were homogenized and incubated in 1 M NaOH containing 10% DMSO at 80 °C for 2 h to measure the melanin content [38]. Then the homogenate were centrifuged and the absorbance of supernate was read at 490 nm. The relative melanin content was calculated as follows:

*Relative melanin = Absorbance (treatment) / Absorbance (Ctrl) × 100%.*

Animal activity and body weight were monitored during the entire experiment period to assess acute toxicity. The liver and lung of each mouse were fixed by formalin and examined by hematoxylin-eosin (HE) staining.

### Statistical analysis

Each experiment was repeated at least three times and the data were presented as mean ± standard deviation (SD). Statistical analyses were performed with SPSS software (version 17.0, SPSS Inc., Chicago, IL, USA). One-way analysis of variance (ANOVA) was used to determine statistical differences between multiple groups. *P* < 0.05 was considered to be statistically significant.

## Results

### Inhibitory effect of J-4 on PKCζ and cell viability

**Fig. 1:**
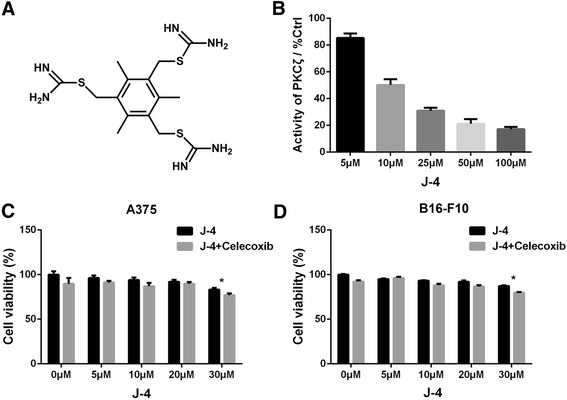
The inhibition of J-4 on PKCζ activity and melanoma cells viability. (**a**) Molecular structure of J-4. (**b**) The inhibitory effect of J-4 on PKCζ activity evaluated by the Z’-LYTE™ KINASE ASSAY KIT-SER/THR 7 PEPTIDE kit. (**c** and **d**) The cell viability of A375 (**c**) and B16-F10 (**d**) were slightly affected by a 24-h treatment of J-4, Celecoxib (25 μM) or their combination measured by MTT assay. * P < 0.05

J-4 is an effective small-molecule inhibitor of PKCζ screened via a Z’-LYTE™ KINASE ASSAY KIT and its molecular structure is shown in Fig. 1A. J4 inhibited the activity of PKCζ in a dose-dependent manner, and its half maximal inhibitory concentration (IC_50_) was calculated to be about 10 μM (Fig. 1B), which was consistent with previous reports [35]. MTT assays revealed that J-4 alone or combined with Celecoxib had slightly influence on viability of A375 (Fig. 1C) and B16-F10 (Fig. 1D) cells at indicated doses, which would not interfere with the following assays of motility properties.

### J-4 combined with celecoxib synergistically inhibited melanoma cells invasion

**Fig. 2:**
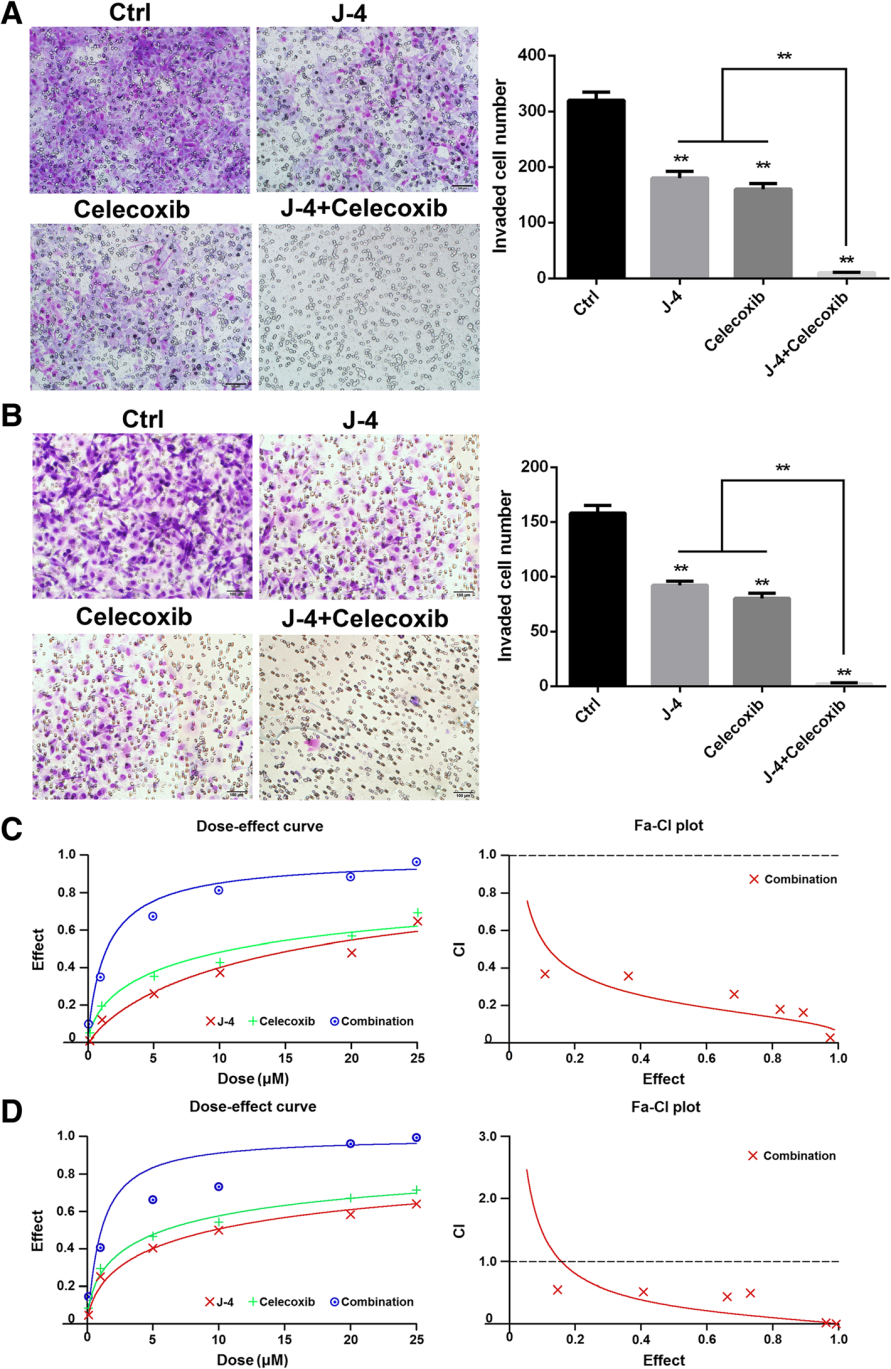
Combined treatment of J-4 and Celecoxib synergistically inhibited the invasion of melanoma cells. (**a** and **b**) The invasion of B16-F10 (**a**) and A375 (**b**) cells was significantly inhibited by a 24-h treatment of the combination of J-4 (25 μM) and Celecoxib (25 μM) assessed via Transwell assay. (**c** and **d**) The dose-effect curve and CI of the synergistic effect of J-4 with Celecoxib in A375 (**c**) and B16-F10 (**d**) cells calculated by the *CalcuSyn software 2.1*. * P < 0.05; ** P < 0.01

Cell invasion is a critical step in cancer metastasis. To investigate the synergistic effects of J-4 combined with Celecoxib on the invasion of melanoma cells, the Transwell assay was performed. The cells were treated with J-4 (0.1, 1, 5, 10, 20 and 25 μM), Celecoxib (0.1, 1, 5, 10, 20 and 25 μM) and their combination (1:1), respectively. The results of J-4 (25 μM) combined with Celecoxib (25 μM) were shown, which significantly enhanced capability for suppressing the invasion of B16-F10 (Fig. 2A) and A375 (Fig. 2B) cells compared with mono-treatments with J4 or Celecoxib. The dose-effect curve and CI in A375 (Fig. 2C) and B16-F10 cells (Fig. 2D) were calculated by *CalcuSyn software 2.1* according to previous reports [39]*.* The CI at various doses was less than 1, indicating a synergistic effect in the combination of J-4 and Celecoxib.

### J-4 combined with celecoxib severely inhibited melanoma cells migration

**Fig. 3:**
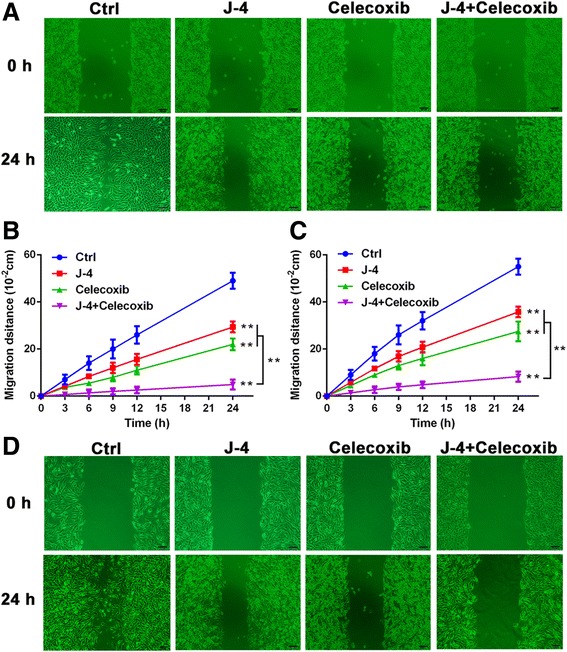
The combination of J-4 and Celecoxib significantly inhibited the migration of melanoma cells. (**a** and **b**) Wound healing assay results in B16-F10 cells with various treatments for 3, 6, 9, 12, and 24 h. (**c** and **d**) Wound healing assay results in A375 cells with various treatments for 3, 6, 9, 12, and 24 h. The migration distance was measured by a software-based method. J-4: 25 μM; Celecoxib: 25 μM. * P < 0.05; ** P < 0.01

The migration of B16-F10 and A375 cells were evaluated using the Wound-healing assay. Compared with control or mono-treatment with J-4 (25 μM) or Celecoxib (25 μM), co-treatment exhibited more potent inhibitory effect on cell migration in B16-F10 (Fig. 3A, B) and A375 cells (Fig. 3C, D). Little mobile was observed with combined treatment after the scratch wound had been healed in control group. The striking differences in the migration distances indicated that the combination of J-4 and Celecoxib severely inhibited the migration of melanoma cells.

### J-4 combined with celecoxib influence cell adhesion and actin polymerization

**Fig. 4:**
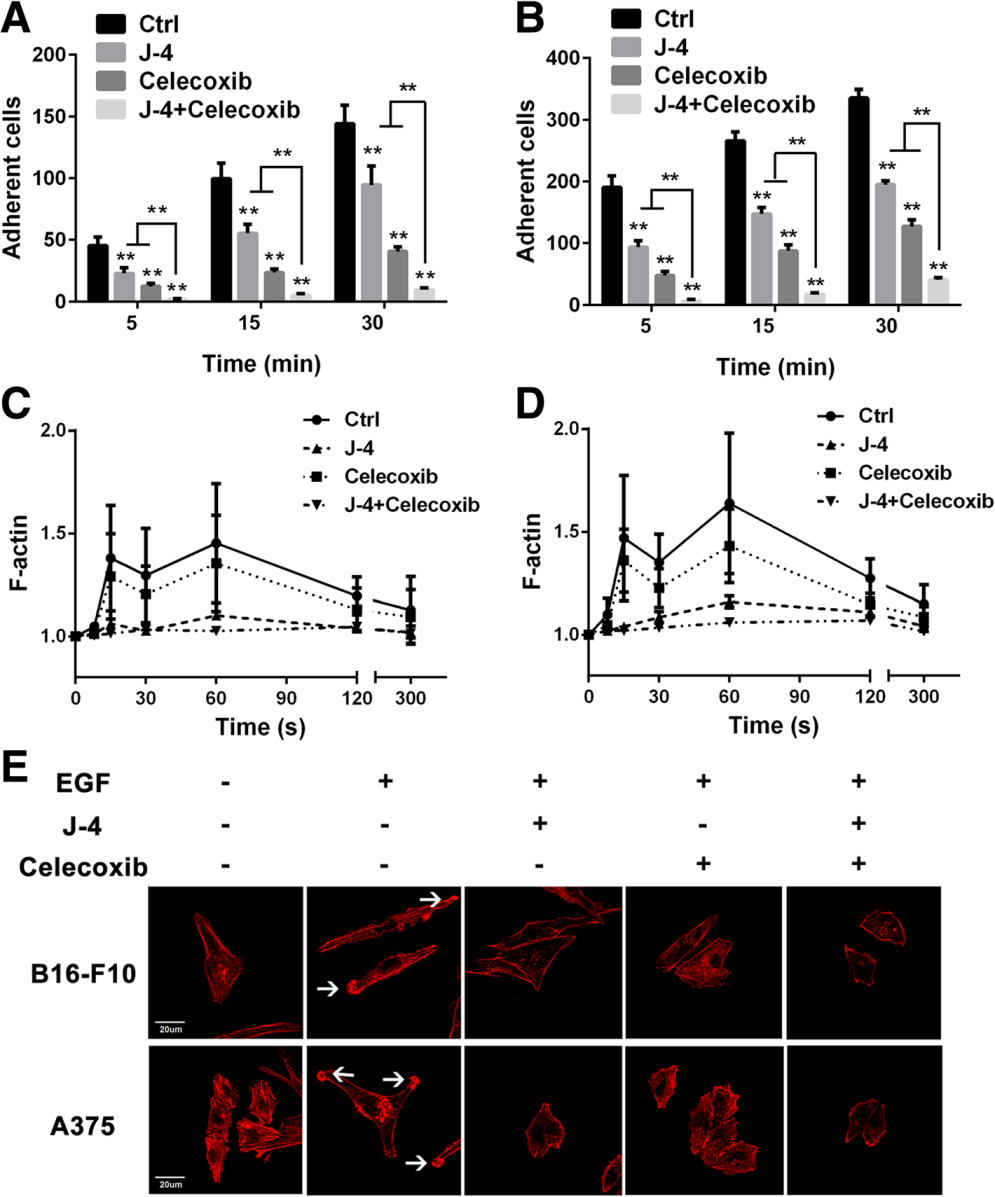
The combination of J-4 and Celecoxib severely affected melanoma cells adhesion and F-actin formation. (**a** and **b**) Adhesion assay results in B16-F10 (**a**) and A375 (**b**) cells at 5, 15 and 30 min after various treatments for 24 h. Cell numbers in five fields were counted for each coverlip under the microscopy with 200 × magnitudes. (**c** and **d**) 20 ng/ml of EGF induced F-actin formation in B16-F10 (**c**) and A375 (**d**) cells were severely inhibited by the combination of J-4 and Celecoxib. (**e**) Confocal images of B16-F10 and A375 cells after various treatments. F-actin was stained with rhodamine labeled phalloidin. EGF: 20 ng/ml; J-4: 25 μM; Celecoxib: 25 μM. * P < 0.05; ** P < 0.01

Cell chemotaxis depends on cell adhesion and actin polymerization. Adhesion assays were performed to assess the effect of J-4 combined with Celecoxib on melanoma cells adhesion. Although treatment with J-4 and/or Celecoxib resulted in a marked reduction in numbers of adherent cells after EGF stimulated for 5, 15 and 30 min, J-4 combined with Celecoxib exhibited more significant inhibition than mono-treatment with J-4 or Celecoxib (Fig. 4A, B). EGF induced actin polymerization was determined by F-actin content and LSCM based immunofluorescence. As shown in Fig. 4C, D, mono-treatment with Celecoxib had slightly influence on EGF induced F-actin formation. When Celecoxib combined with J-4, the two phase peaks of actin polymerization at 15 s and 60s, depending on Cofilin and PI3K [26], respectively, were eliminated. As observed by LSCM (Fig. 4E), F-actin accumulated at the cell leading edges, which further caused the deformations of B16-F10 and A375 cells under the stimulation of EGF. However, the phenomena of F-actin accumulation and cell deformation almost disappeared both in B16-F10 and A375 cells after exposure to combined treatment for 24 h. Taken together, J-4 combined with Celecoxib severely impaired cell adhesion and actin polymerization during melanoma cells motility.

### J-4 combined with celecoxib affect expressions of COX-2 and activities of PKCζ in melanoma cells

**Fig. 5:**
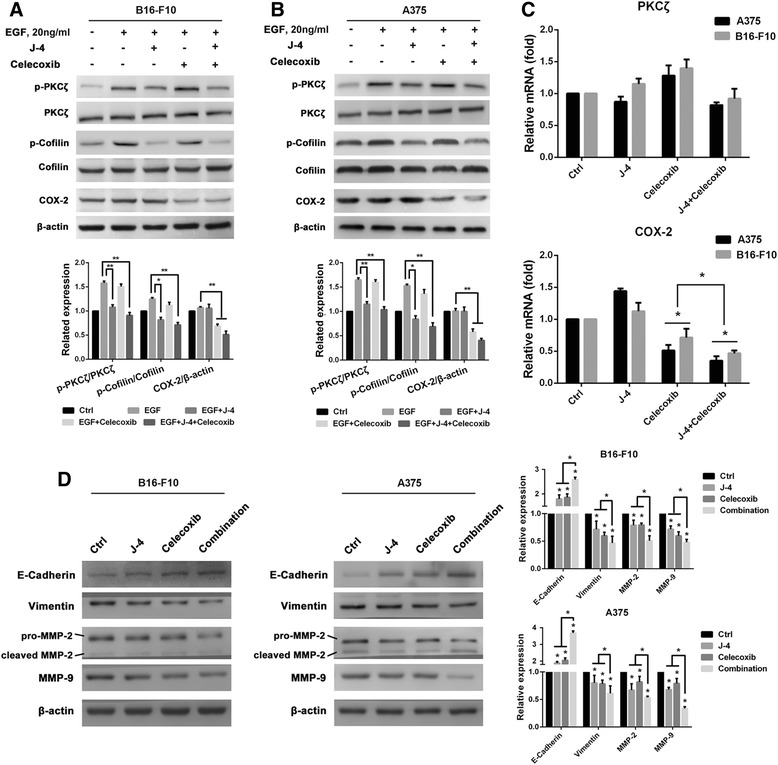
The expression of p-PKCζ, p-cofilin and COX-2 after combined treatment of J-4 and Celecoxib. (**a**) Western blotting images of p-cofilin and COX-2 in B16-F10 cells with various treatments for 24 h. (**b**) Western blotting images of p-cofilin and COX-2 in A375 cells with various treatments for 24 h. (**c**) Relative mRNA level of PKCζ and COX-2 determined via RT-PCR. (**d**) The expression of EMT markers, E-Cadherin and Vimentin, and MMP-2/MMP-9 was affected in B16-F10 and A375 cells after various treatments for 24 h. J-4: 25 μM; Celecoxib: 25 μM. * P < 0.05; ** P < 0.01

To confirm J-4 combined with Celecoxib suppress melanoma cells chemotaxis in a PKCζ and COX-2 dependent manner, Western blotting assays were performed to analyze the expression of p-PKCζ, p-cofilin and COX-2 under EGF stimulation. Cofilin, an actin binding protein, plays an important role in actin polymerization and serves as an indicator of PKCζ related signal pathway. As shown in Fig. 5A, B, mono-treatment of J-4 decreased the phosphorylation of PKCζ and Cofilin induced by EGF, while Celecoxib reduced the over-expression of COX-2. However, co-treatment simultaneously reduced their expressions more significant than J-4 or Celecoxib, suggesting a synergistic but not additive effect existed in the combination of J-4 and Celecoxib. In RT-PCR results (Fig. 5C), total mRNA of PKCζ had no significant variation, indicating combined treatment affected the activity rather than expression of PKCζ. Total mRNA of COX-2 with co-treatment declined more than mono-treatment with Celecoxib both in B16-F10 and A375 cells, suggesting J-4 enhanced the inhibitory effect of Celecoxib on COX-2. Taken together, J-4 combined with Celecoxib synergistically suppressed the activity of PKCζ and expression of COX-2. In addition, after treatment with the combination of J-4 (25 μM) and Celecoxib (25 μM), the expression of E-Cadherin increased more than 2- and 3-fold in B16-F10 and A375 cells, respectively, while the expression of Vimentin both decreased about 50% in the two cell lines (Fig. 5D). As reported previously [40, 41], PKCζ plays an important role in the secretion of MMP-2/MMP-9 and the results of mono-treatment of J-4 further support it. However, co-treatment with J-4 and Celecoxib decreased the expression of MMP-2/MMP-9 more than each mono-treatment (Fig. 5D). The results indicate the combination of two inhibitors could induce MET in melanoma cells and decrease the expression of MMP-2/MMP-9.

### J-4 and celecoxib blocked melanoma lung metastasis in vivo

**Fig. 6:**
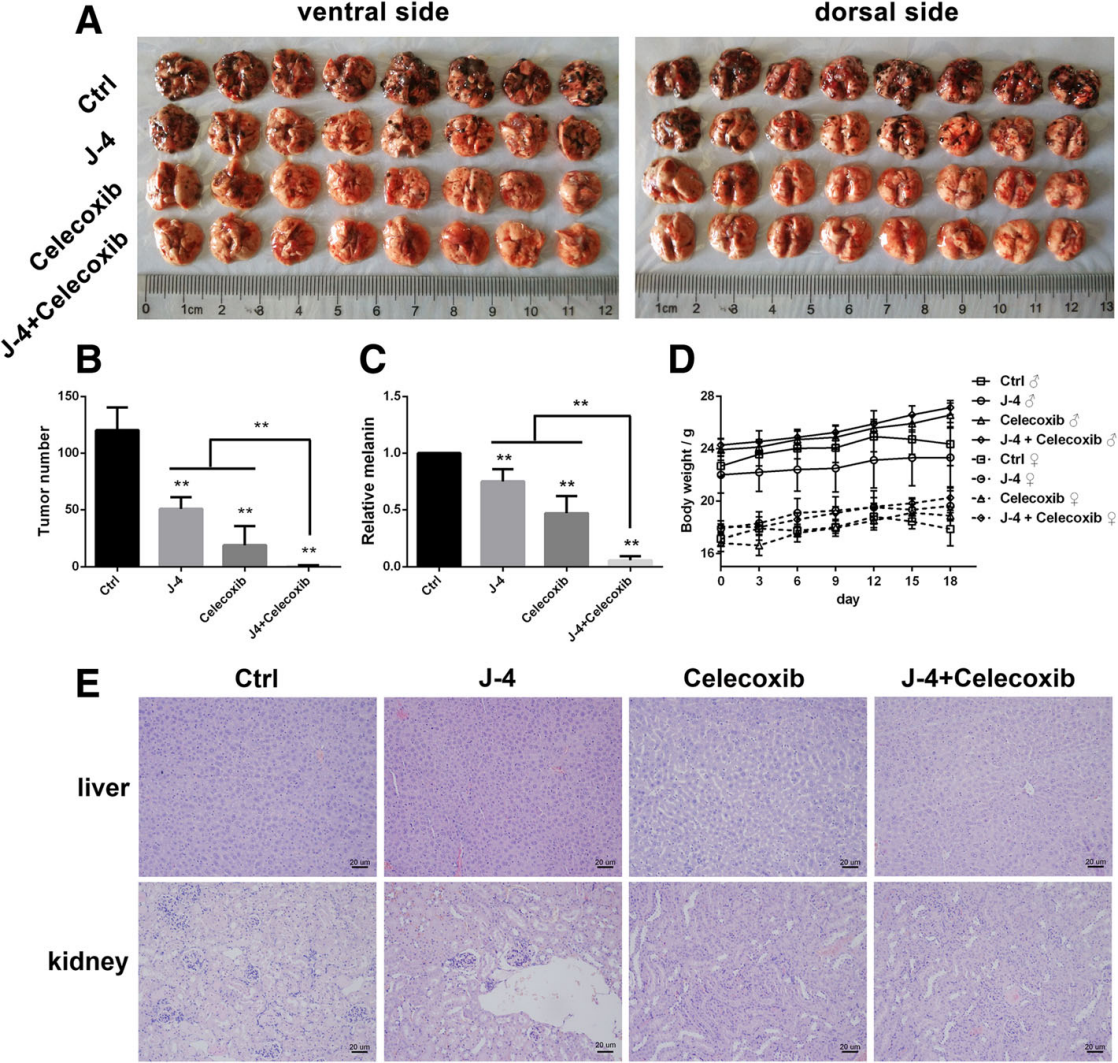
In vivo study of the combination therapy of J-4 and Celecoxib via B16-F10/C57BL mouse melanoma model. (**a**). Ventral and dorsal photograph of lungs removed from the mice after various treatments. (**b**) Comparison of lung metastatic notes in mice with various treatments. (**c**) The relative melanin content of lung homogenate. (**d**) The body weight changes of the mice during the treatment period. (**e**) HE staining results of livers and kidneys (100×). * P < 0.05; ** P < 0.01

B16-F10/C57BL mouse melanoma lung metastasis model is a classic method to evaluate cell metastasis capability in vivo [42, 43]. In order to test the efficacy of J-4 combined with Celecoxib in preventing tumor lung metastasis, B16-F10 cells were intravenously injected into C57BL/6 mice, which were then treated with J-4 (20 mg/kg), Celecoxib (20 mg/kg) and their combination for 3 weeks, respectively. Compared with control and mono-treatments, co-treatment with J-4 and Celecoxib exhibited more efficient in reducing pulmonary metastatic nodules and almost blocked melanoma lung metastasis (Fig. 6A, B). The result was further confirmed by the melanin content determination (Fig. 6C). No notable variation of body weight was observed during the entire experiment period (Fig. 6D), suggesting that co-treatment with J-4 and Celecoxib was low toxic in mammals. To further confirm the safety of the combination therapy, the liver and kidney of each mouse was analyzed by HE staining. No necrosis was observed in J-4, Celecoxib or their combination group (Fig. 6E). All above results signified that J-4 combined with Celecoxib possessed potent inhibitory effects on cancer metastasis but showed no significant toxicity.

## Discussion

The advanced and metastasized melanoma always indicates poor survival and lacks effective drugs in clinic [3]. Cancer metastasis is a complicated event that involves multiple sequential and interlinked steps including detachment, migration, invasion and adhesion. Thus, drug combination is reasonably raised as a promising strategy for melanoma metastasis [5]. The activity of PKCζ and expression of COX-2 are two essential elements for melanoma metastasis, since cell chemotaxis is mediated by PKCζ and COX-2 dependent signaling pathways. In this study, the inhibitory capability of combined inhibition of PKCζ and COX-2 by their inhibitors J-4 and Celecoxib, respectively, was evaluated both in vitro and in vivo. J-4 is a small-molecule inhibitor specific for PKCζ screened by our group with IC50 at approximately 10 μM and Celecoxib is a highly selective inhibitor of COX-2 which has been widely tested in clinical trials for treatment of many types of cancer. Co-treatment with J-4 and Celecoxib in A375 and B16 cells did not significantly affect cell proliferation, but severely impaired cell migration, invasion and adhesion which were all required for melanoma cells motility. The results are consistent with the phenotype induced by PKCζ or COX-2 inhibition in previous reports [19, 35], which means cell motility inhibition but not cell death [44]. The CI value is a widely accepted indicator of synergistic effect [39]. The CI calculated by *CalcuSyn software 2.1* signifies that J-4 combined with Celecoxib is synergistic rather than additive effect.

PKCζ and COX-2 related pathways play an important role in EGF induced cell chemotaxis [24]. The phosphorylation of PKCζ and Cofilin serve as main indicators of PKCζ activity [23] and the function of COX-2 depends on its expression [11]. J-4 severely decreased the phosphorylation of PKCζ and Cofilin under EGF stimulation without affecting their expressions and COX-2, while Celecoxib reduced the expression of COX-2 both at protein and mRNA levels without affecting the activity of PKCζ. However, co-treatment with J-4 and Celecoxib induced more significant decrease than mono-treatments, further supporting the combination is synergistic effect. The results also indicate J-4 combined with Celecoxib suppresses cell motility via impairing the activity of PKCζ and the expression of COX-2. Cell migration depends on F-actin aggregation at the cell leading edges and further induced formation of lamellipodia [24]. After co-treatment with J-4 and Celecoxib, EGF induced F-actin aggregation disappeared, which correlated to the dephosphorylation of Cofilin and suggested the inactivation of PKCζ related pathways. In addition, the combination of J-4 and Celecoxib could induce MET and decrease the expression of MMP-2/MMP-9 in melanoma cells, which in turn inhibit the migration and invasion of melanoma cells.

Melanoma is highly metastatic, and lung is one of the major target organs for metastasis. B16-F10/C57BL mouse melanoma lung metastasis model is widely used to screen drugs for cancer metastasis in preclinical trials [42, 43] and B16-F10 is a highly lung metastatic cell line screened from B16 cells [45]. In this study, co-treatment with J-4 and Celecoxib almost blocked the lung metastasis of the intravenously injected B16-F10 cells. Furthermore, no notable variation of animal activities and body weights were observed during the entire experiment period, indicating low toxicity of the therapy, which was further confirmed by HE staining results.

## Conclusion

In conclusion, combination therapy of PKCζ and COX-2 inhibitors could synergistically suppress melanoma cells migration, invasion and adhesion in vitro and block melanoma lung metastasis in vivo. It represents a novel and promising therapy for advanced and metastasized melanoma with low toxicity.

## Abbreviations

CI: combination index
FBS: fetal bovine serum
HE: hematoxylin-eosin
IC_50_: half maximal inhibitory concentration
LSCM: laser scanning confocal microscope
MTT: Methylthiazolyldiphenyl-tetrazolium bromide
PKCζ: protein kinase C ζ COX-2 cyclooxygenase-2
RT-PCR: Real time PCR

## References

[1] Dunn J, Watson M (2016). Aitken JF.

[2] Park SM, Li T, Wu S, Li WQ, Weinstock M, Qureshi AA, Cho E (2017). Niacin intake and risk of skin cancer in US women and men. Int J Cancer.

[3] Loquai C, Schmidtmann I, Garzarolli M, Kaatz M, Kahler KC, Kurschat P, Meiss F, Micke O, Muecke R, Muenstedt K, et al. Interactions from complementary and alternative medicine in patients with melanoma. Melanoma Res. 2017; in press 10.1097/CMR.0000000000000339 28252553

[4] Chhabra A, Mukherji B, Batra D (2017). Activation induced cell death (AICD) of human melanoma antigen-specific TCR engineered CD8 T cells involves JNK, Bim and p53. Expert Opin Ther Targets.

[5] Polkowska M, Czepielewska E, Kozlowska-Wojciechowska M (2016). Drug combinations as the new standard for melanoma treatment. Curr Treat Options in Oncol.

[6] Madeddu C, Dessi M, Panzone F, Serpe R, Antoni G, Cau MC, Montaldo L, Mela Q, Mura M, Astara G (2012). Randomized phase III clinical trial of a combined treatment with carnitine plus celecoxib +/− megestrol acetate for patients with cancer-related anorexia/cachexia syndrome. Clin Nutr.

[7] Li Z, Ye S, OuYang L, Zhang H, Chen Y, He J, Chen Q, Qian C, Zhang X, Cui J, et al: COX-2 promotes metastasis in nasopharyngeal carcinoma by mediating interactions between cancer cells and myeloid-derived suppressor cells. *Oncoimmunology* 2015, **4:**e1044712. eCollection. 10.1080/2162402X.2015.1044712 PMC4590030 26451317

[8] Guo Z, Jiang J, Zhang J, Yang H, Yang F, Qi Y, Zhong Y, Su J, Yang R, Li L, Xiang B (2015). COX-2 promotes migration and invasion by the side population of cancer stem cell-like hepatocellular carcinoma cells. Medicine.

[9] Cao C, Gao R, Zhang M, Amelio AL, Fallahi M, Chen Z, Gu Y, Hu C, Welsh EA, Engel BE (2015). Role of LKB1-CRTC1 on glycosylated COX-2 and response to COX-2 inhibition in lung cancer. J Natl Cancer Inst.

[10] Kim KM, Im A, Kim SH, Hyun JW, Chae S (2016). Timosaponin AIII inhibits melanoma cell migration by suppressing COX-2 and in vivo tumor metastasis. Cancer Sci.

[11] Goulet AC, Einsphar JG, Alberts DS, Beas A, Burk C, Bhattacharyya A, Bangert J, Harmon JM, Fujiwara H, Koki A, Nelson MA (2003). Analysis of cyclooxygenase 2 (COX-2) expression during malignant melanoma progression. Cancer Biol Ther.

[12] Panza E, De Cicco P, Ercolano G, Armogida C, Scognamiglio G, Anniciello AM, Botti G, Cirino G, Ianaro A (2016). Differential expression of cyclooxygenase-2 in metastatic melanoma affects progression free survival. Oncotarget.

[13] Szymanski PT, Muley P, Ahmed SA, Khalifa S, Fahmy H (2012). Sarcophine-diol inhibits expression of COX-2, inhibits activity of cPLA2, enhances degradation of PLA2 and PLC(gamma)1 and inhibits cell membrane permeability in mouse melanoma B16F10 cells. Mar Drugs.

[14] Adler NR, Haydon A, McLean CA, Kelly JW, Mar VJ (2017). Metastatic pathways in patients with cutaneous melanoma. Pigment Cell Melanoma Res.

[15] El-Rayes BF, Zalupski MM, Shields AF, Ferris AM, Vaishampayan U, Heilbrun LK, Venkatramanamoorthy R, Adsay V, Philip PA (2005). A phase II study of celecoxib, gemcitabine, and cisplatin in advanced pancreatic cancer. Invest New Drug.

[16] Elmets CA, Viner JL, Pentland AP, Cantrell W, Lin H, Bailey H, Kang S, Linden KG, Heffernan M, Duvic M (2010). Chemoprevention of nonmelanoma skin cancer with celecoxib: a randomized, double-blind, placebo-controlled trial. J Natl Cancer Inst.

[17] Javle MM, Cao S, Durrani FA, Pendyala L, Lawrence DD, Smith PF, Creaven PJ, Noel DC, Iyer RV, Rustum YM (2007). Celecoxib and mucosal protection: translation from an animal model to a phase I clinical trial of celecoxib, irinotecan, and 5-fluorouracil. Clin Cancer Res.

[18] Seo KW, Coh YR, Rebhun RB, Ahn JO, Han SM, Lee HW, Youn HY (2014). Antitumor effects of celecoxib in COX-2 expressing and non-expressing canine melanoma cell lines. Res Vet Sci.

[19] Sadhu SS, Wang S, Averineni RK, Seefeldt T, Yang Y, Guan X (2016). In-vitro and in-vivo inhibition of melanoma growth and metastasis by the drug combination of celecoxib and dacarbazine. Melanoma Res.

[20] Gowda R, Sharma A, Robertson GP (2017). Synergistic inhibitory effects of celecoxib and Plumbagin on melanoma tumor growth. Cancer Lett.

[21] Niessner H, Sinnberg T, Kosnopfel C, Smalley KSM, Beck D, Praetorius C, Mai M, Beissert S, Kulms D, Schaller M, et al. BRAF inhibitors amplify the pro-apoptotic activity of MEK inhibitors by inducing ER stress in NRAS-mutant melanoma. Clin Cancer Res. 2017; 10.1158/1078-0432.CCR-17-0098. in press. 10.1158/1078-0432.CCR-17-0098 PMC5641247 28724666

[22] Escuin-Ordinas H, Atefi M, Fu Y, Cass A, Ng C, Huang RR, Yashar S, Comin-Anduix B, Avramis E, Cochran AJ (2014). COX-2 inhibition prevents the appearance of cutaneous squamous cell carcinomas accelerated by BRAF inhibitors. Mol Oncol.

[23] Guo H, Ma Y, Zhang B, Sun B, Niu R, Ying G, Zhang N (2009). Pivotal advance: PKC zeta is required for migration of macrophages. J Leukoc Biol.

[24] Sun RH, Gao P, Chen L, Ma DL, Wang JM, Oppenheim JJ, Zhang N (2005). Protein kinase C zeta is required for epidermal growth factor-induced chemotaxis of human breast cancer cells. Cancer Res.

[25] Schondorf T, Kurbacher CM, Becker M, Warm M, Kolhagen H, Gohring UJ (2001). Heterogeneity of proteinkinase C activity and PKC-zeta expression in clinical breast carcinomas. Clin Exp Med.

[26] Guo H, Gu F, Li W, Zhang B, Niu R, Fu L, Zhang N, Ma Y (2009). Reduction of protein kinase C zeta inhibits migration and invasion of human glioblastoma cells. J Neurochem.

[27] Liu Y, Wang B, Wang J, Wan W, Sun R, Zhao Y, Zhang N (2009). Down-regulation of PKCzeta expression inhibits chemotaxis signal transduction in human lung cancer cells. Lung Cancer.

[28] Krasagakis K, Fimmel S, Genten D, Eberle J, Quas P, Ziegler W, Haller H, Orfanos CE (2002). Lack of protein kinase C (PKC)-beta and low PKC-alpha, −delta, −epsilon, and -zeta isozyme levels in proliferating human melanoma cells. Int J Oncol.

[29] Certa U, Seiler M, Padovan E, Spagnoli GC (2002). Interferon-a sensitivity in melanoma cells: detection of potential response marker genes. Recent Results Cancer Res.

[30] Oka M, Kikkawa U (2005). Protein kinase C in melanoma. Cancer Metastasis Rev.

[31] Tsubaki M, Matsuoka H, Yamamoto C, Kato C, Ogaki M, Satou T, Itoh T, Kusunoki T, Tanimori Y, Nishida S (2007). The protein kinase C inhibitor, H7, inhibits tumor cell invasion and metastasis in mouse melanoma via suppression of ERK1/2. Clin Exp Metastasis.

[32] Hodgson L, Henderson AJ, Dong C (2003). Melanoma cell migration to type IV collagen requires activation of NF-kappaB. Oncogene.

[33] Wu J, Liu S, Fan Z, Zhang L, Tian Y, Yang R (2016). A novel and selective inhibitor of PKC zeta potently inhibits human breast cancer metastasis in vitro and in mice. Tumor Biol.

[34] Wu J, Zhang B, Wu M, Li H, Niu R, Ying G, Zhang N (2010). Screening of a PKC zeta-specific kinase inhibitor PKCzI257.3 which inhibits EGF-induced breast cancer cell chemotaxis. Invest New Drug.

[35] Li H, Wu J, Ying G, Chen L, Lai L, Liu Z, Zhang N, Guo H (2012). J-4: a novel and typical preclinical anticancer drug targeting protein kinase C zeta. Anti-Cancer Drug.

[36] Lu S, Smith AP, Moore D, Lee NM (2010). Different real-time PCR systems yield different gene expression values. Mol Cell Probes.

[37] Duary RK, Bhausaheb MA, Batish VK, Grover S (2012). Anti-inflammatory and immunomodulatory efficacy of indigenous probiotic lactobacillus plantarum Lp91 in colitis mouse model. Mol Biol Rep.

[38] Chang MS, Choi MJ, Park SY, Park SK (2010). Inhibitory effects of Hoelen extract on Melanogenesis in B16/F1 melanoma cells. Phytother Res.

[39] Zhang N, Fu J, Chou T (2016). Synergistic combination of microtubule targeting anticancer fludelone with cytoprotective panaxytriol derived from panax ginseng against MX-1 cells in vitro: experimental design and data analysis using the combination index method. Am J Cancer Res.

[40] Hussain S, Assender JW, Bond M, Wong LF, Murphy D, Newby AC (2002). Activation of protein kinase Czeta is essential for cytokine-induced metalloproteinase-1, −3, and −9 secretion from rabbit smooth muscle cells and inhibits proliferation. J Biol Chem.

[41] Goichberg P, Kalinkovich A, Borodovsky N, Tesio M, Petit I, Nagler A, Hardan I, Lapidot T (2006). cAMP-induced PKCzeta activation increases functional CXCR4 expression on human CD34+ hematopoietic progenitors. Blood.

[42] Wang Z, Chen J, Wang J, Ahn S, Li C, Lu Y, Loveless VS, Dalton JT, Miller DD, Li W (2012). Novel tubulin polymerization inhibitors overcome multidrug resistance and reduce melanoma lung metastasis. Pharm Res-Dordr.

[43] Amarzguioui M, Peng Q, Wiiger MT, Vasovic V, Babaie E, Holen T, Nesland JM, Prydz H (2006). Ex vivo and in vivo delivery of anti-tissue factor short interfering RNA inhibits mouse pulmonary metastasis of B16 melanoma cells. Clin Cancer Res.

[44] Bell E, Ponthan F, Whitworth C, Tweddle DA, Lunec J, Redfern CPF (2014). COX2 expression in neuroblastoma increases tumorigenicity but does not affect cell death in response to the COX2 inhibitor celecoxib. Clin Exp Metastas.

[45] Kim SH, Kim Y, Kim M, Kim DS, Lee SC, Chi S, Lee DH, Park SG, Park BC, Bae K, Kang S (2009). Comparative proteomic analysis of mouse melanoma cell line B16, a metastatic descendant B16F10, and B16 overexpressing the metastasis-associated tyrosine phosphatase PRL-3. Oncol Res.

