# Correction to: Combination therapy of PKCζ and COX-2 inhibitors synergistically suppress melanoma metastasis

**DOI:** 10.1186/s13046-021-01885-y

**Published:** 2021-03-01

**Authors:** Ping Zhou, Jiaqi Qin, Yuan Li, Guoxia Li, Yinsong Wang, Ning Zhang, Peng Chen, Chunyu Li

**Affiliations:** Department of Thoracic Medical Oncology, Tianjin Medical University Cancer Institute and Hospital, School of Basic Medical Sciences, International Medical School, School of Pharmacy, Tianjin Medical University, No. 22 Qixiangtai Road, Heping District, Tianjin, 300070 People’s Republic of China

**Correction to: J Exp Clin Cancer Res 36, 115 (2017)**

**https://doi.org/10.1186/s13046-017-0585-2**

Following publication of the original article [1], the authors identified some minor errors in image-typesetting in Figs. 2 and 3; specifically in Fig. 2b, and Fig. 3a and d.

**Fig. 2:**
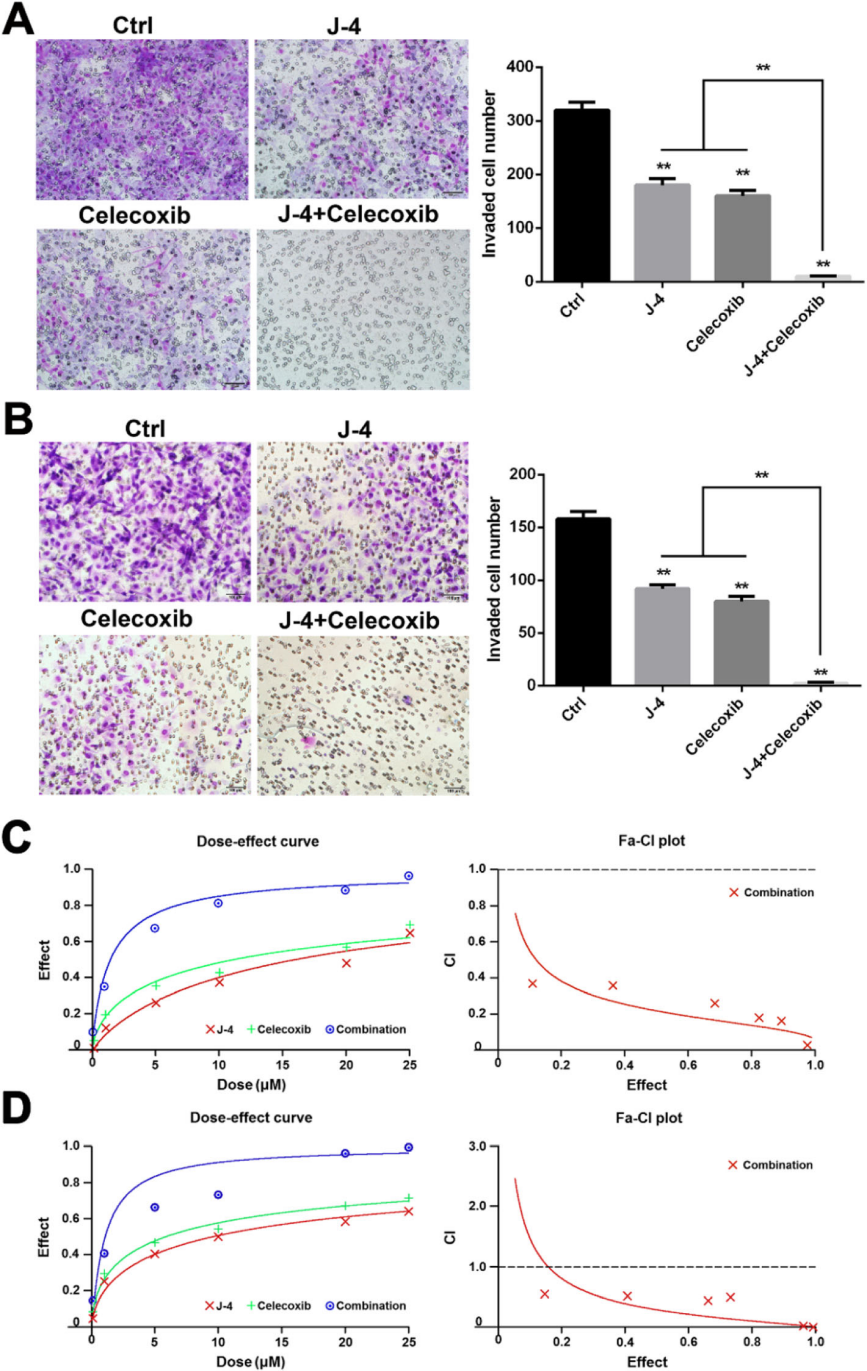
Combined treatment of J-4 and Celecoxib synergistically inhibited the invasion of melanoma cells. **a** and **b** The invasion of B16-F10 (**a**) and A375 (**b**) cells was significantly inhibited by a 24-h treatment of the combination of J-4 (25 μM) and Celecoxib (25 μM) assessed via Transwell assay. **c** and **d** The dose-effect curve and CI of the synergistic effect of J-4 with Celecoxib in A375 (**c**) and B16-F10 (**d**) cells calculated by the CalcuSyn software 2.1. * *P* < 0.05; ** *P* < 0.0

**Fig. 3:**
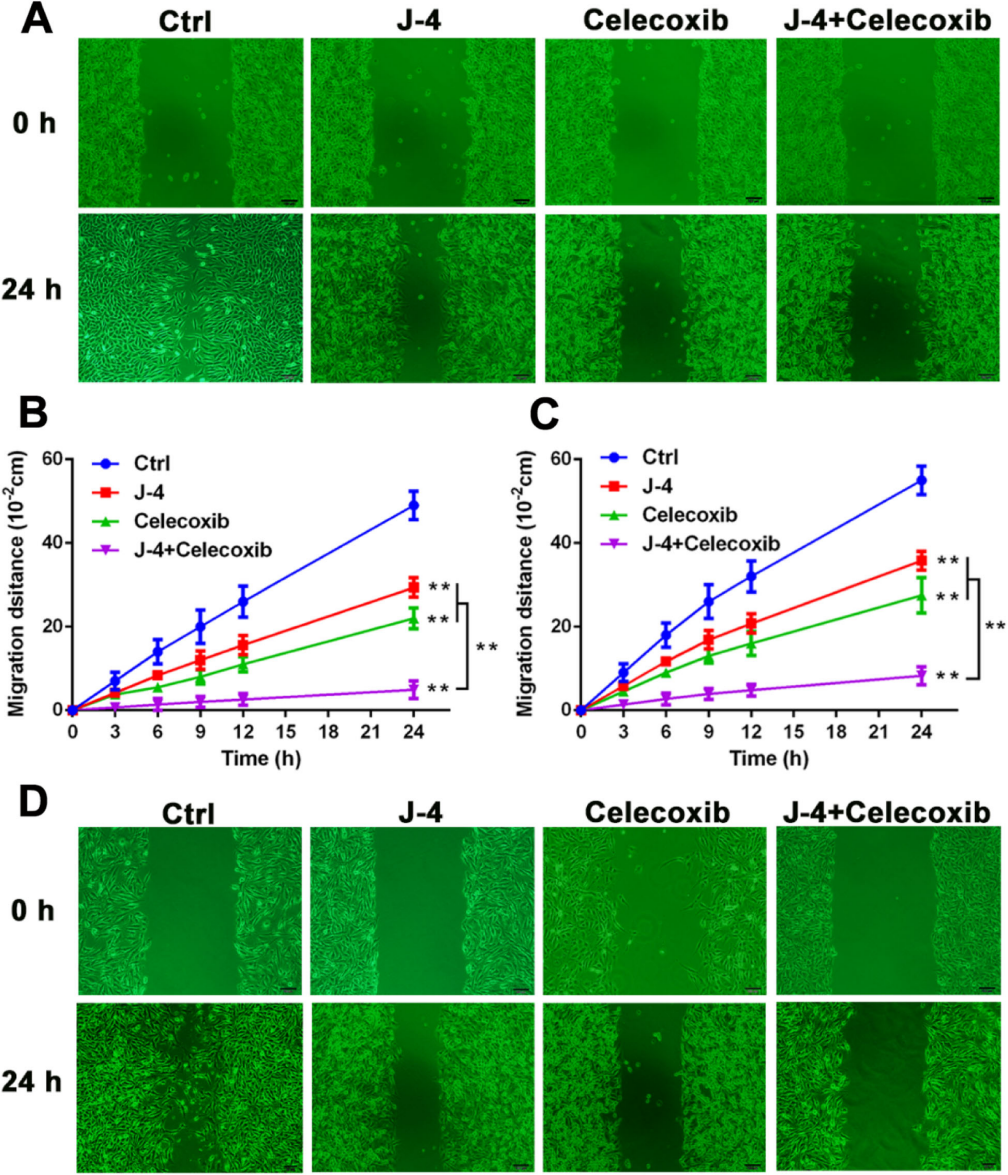
The combination of J-4 and Celecoxib significantly inhibited the migration of melanoma cells. **a** and **b** Wound healing assay results in B16-F10 cells with various treatments for 3, 6, 9, 12, and 24 h. **c** and **d** Wound healing assay results in A375 cells with various treatments for 3, 6, 9, 12, and 24 h. The migration distance was measured by a software-based method. J-4: 25 μM; Celecoxib: 25 μM. * *P* < 0.05; ** *P* < 0.01

In Fig. 2b, the picture of cell invasion assay in J-4 group has been corrected.

In Fig. 3a, the picture of wound healing assay in J-4 group (24 h) has been corrected.

In Fig. 3d, the pictures of wound healing assay in Celecoxib group (0 h) has been corrected.

The corrected figures are given below. The corrections do not have any effect on the final conclusions of the paper.
